# Validation of the Weight Bias Internalization Scale for Mainland Chinese Children and Adolescents

**DOI:** 10.3389/fpsyg.2020.594949

**Published:** 2021-01-06

**Authors:** Hao Chen, Yi-duo Ye

**Affiliations:** School of Psychology, Fujian Normal University, Fuzhou, China

**Keywords:** weight stigma internalization, Chinese adolescents, item response theory, psychometric properties, classical test theory

## Abstract

Weight stigma internalization among adolescents across weight categories leads to adverse psychological consequences. This study aims to adapt and validate a Chinese version of the Weight Bias Internalization Scale for Mainland Chinese children and adolescents(C-WBIS). A total of 464 individuals aged 9 to 15 years participated in the present study. Based on item response theory (IRT) and classical test theory (CTT), we selected the items for the C-WBIS and evaluated its reliability and validity. The item response theory yields support for the one-dimensional factor mode. All item parameters fit the IRT model (albeit within an adequate range), eight items were adopted. No evidence of significant differential item functioning (DIF) was found for gender and age groups. The C-WBIS was correlated with the Core Self-Evaluation Scale (CSES) and two subscales of the Social Anxiety Scale for Children (SAS), which indicated an acceptable criterion-related validity. The C-WBIS is a reliable and valid measure that can be used as a psychometrically sound and informative tool to assess weight bias internalization among children and adolescents.

## Introduction

There has been a considerable increase in research on the stigma toward individuals of varying weight statuses since Goffman’s pioneering work (Goffman, 1968). Experiences of weight bias related to weight bias internalization among children are conceived as a highly prevalent issue in most countries (Pearl and Puhl, 2018). Weight bias internalization (WBI) is defined as internalizing the reactions of discriminatory groups and enacting the stereotypes toward individual with abnormal weight, leading to negative emotional reactions (Pearl et al., 2015). It occurs when individuals apply negative weight stereotypes (e.g., slothfulness) to themselves (Pearl, 2018). It is operationalized as self-devaluation or self-directed stigma (Tomiyama, 2014), and correlational evidence suggests that it affects individuals of varying weight statuses (i.e., underweight, normal, and overweight individuals) (Schvey and White, 2015), which is not limited to overweight and obese individuals. Thus, those with a normal weight also internalize these negative stereotypes (Kurth and Ellert, 2008). It substantially influences psychological outcomes as an experience of weight-based stigmatization enacted by others, and leads to greater negative affect as well as lower self-esteem (Pearl and Puhl, 2016; Pearl et al., 2018).

Prior research on self-reported measures for assessing self-stigma suggests that studies predominantly use the Weight Self-Stigma Questionnaire(WSSQ) for overweight and obese individuals and the Weight Bias Internalization Scale(WBIS) across weight categories among children and adults. The WSSQ consists of two underlying psychological dimensions using (a) self-devaluation and (b) fear of enacted stigma (Lillis et al., 2010; Maïano et al., 2019). The WBIS provides a concise measure of weight bias internalization, suggesting that weight bias internalization is a distinct construct from anti-fat attitudes, self-esteem, and body image (Durso and Latner, 2008). Meanwhile, the WBIS has excellent psychometric properties and construct validity, as it has been widely used for among adults (Hilbert et al., 2014a; Pearl and Puhl, 2014) and school-aged children (Anna and Petra, 2018).

Contemporary literature indicates that the WBIS has been validated as a psychometrically sound and informative tool to assess weight bias internalization and has supported a unidimensional interpretation of the WBIS. More specifically, the original validation of the WBIS has analyzed the 11-item WBIS to measure weight bias internalization, and included a sample of 198 participants aged >18 years and self-identified as overweight. Cronbach’s alpha was 0.90. One-dimensional factors would be retained when the component extraction was set to one. The scale has significant partial correlations with the dislike subscale of the Antifat Attitudes Questionnaire(AAQ) and the drive for thinness in the Eating Disorders Inventory(EDI),suggesting that anti-fat attitudes, drive for thinness and weight bias internalization are related but different from each other (Durso and Latner, 2008).

The revised German version of the WBIS contains 11 items in a large sample of 1092 participants aged between 14 and 89 to conduct the latent variable structure of the WBIS. Cronbach’s alpha was 0.91 after items one was deleted. The goodness-of-fit criteria supported the single-structure of the 10-item WBIS (Hilbert et al., 2014a). To be broadly applicable to individuals of varying weight statuses, the modified WBIS (WBIS-M) contains 11 items with a seven- point Likert frequency response scale in a sample of 148 participants between 19 and 70 years old. Further, it rewords items, starting at “my weight,” whereas the original scale starts at “overweight.” For example, the beginning of item one was changed from “As an overweight person.” to “Because of my weight.” Cronbach’s alpha was 0.94. Moreover, there is an inverse relationship between self-esteem and the WBIS, suggesting that higher levels of weight bias internalization are related to lower self-esteem (Pearl and Puhl, 2014).

Furthermore, the 11 item Weight Bias Internalization Scale for Youth (WBIS-Y) provides a valid and reliable assessment tool in a sample of 191 German adolescents aged between 13 and 19. Items were modified to be adjusted for adolescents. Cronbach’s alpha was 0.87. The first eigenvalue (4.92) explained 44.70% of the variance, and the ratio of the first to the second eigenvalue was 4.0,which supported the single-factor structure of the WBIS (Ciupitu-Plath et al., 2017). A recent study on 1148 school children aged between 9 and 13 across weight categories using the WBIS-M, the Weight Bias Internalization Scale for Children (WBIS-C) explored weight bias internalization among younger children. Cronbach’s alpha was 0.86 after excluding item one. The one-factor model was strictly invariant across gender groups (Anna and Petra, 2018).

Although researchers have made great efforts on the operational definition and empirical measurement, it remains somewhat contested, and raises questions about whether to measure the latent trait that it is associated with weight bias internalization. In addition, studies on the WBIS have been conducted in Western countries. Such studies on school-aged adolescents across weight categories remain under investigation, especially in East Asian regions.

### Measurement of Weight Bias Internalization in China

China is the most populous country in East Asia. Evidently, only a few studies conducted with children or adults have focused on assessing degree of self-stigma, such as perception of incorrect beliefs (Lin and Lee, 2017; Chan et al., 2019; Pakpour et al., 2019; Wong et al., 2019). In a Hong Kong study on 367 children aged between 8 and 12, Chan et al. (2019) found that overweight children had a higher level of self-stigma, which in turn caused poor mental health problems. Meanwhile. Wong et al. (2019) found that overweight children had a higher level of self-stigma, which was associated with poorer health-related quality of life. However, the aforementioned studies merely translated the WBIS into Chinese and did not report Cronbach’s alpha indicated as regards level of reliability for their own study. Thus, content validation of the scale items remain under investigation as the aforementioned studies cannot indicate what necessitated the need for extra revision. Besides, it is generally agreed that the measurement invariance or measurement equivalence are usually explored when conducting subcultural analyses or comparing different groups (Smid et al., 2018). The measurement invariance of the WBIS has been validated among a sample of children and adolescents in Hong Kong to assess weight bias internalization (Pakpour et al., 2019). Whereas, Hong Kong, colonized by Great Britain, has developed distinctive subcultures, leading to different cultural environments from the Chinese mainland (Wu, 2011). Thus, further verification is required to investigate measurement invariance associated with weight bias internalization across weight categories. Such verification would correct the estimates of effects in research in which weight stigma has been shown to be prevalent among youths of any weight (Widaman, 1993; Anna and Petra, 2018). In turn, these results are associated with weight bias internalization and linked to negative health outcomes (Puhl and Heuer, 2010). To our knowledge, there is still insufficient evidence regarding the applicability of the WBIS to Mainland Chinese children and adolescents. This study addressed these aspects for the Chinese version of the WBIS among Mainland Chinese children and adolescents (C-WBIS).

## Psychometric Methods

Previous studies have reported the psychometric properties of the WBIS. However, additional psychometric evaluations are necessary as most of the studies evaluating the psychometric properties of the WBIS merely use classical statistical methods, such as exploratory factor analysis, internal consistency, and test-retest reliability. These methods essentially use the correlation coefficient that falls within “classical test theory” as it has the same underlying weakness as the other methods analyzing correlations among items (Oishi, 2006). These weaknesses are specific to a given sample and result in bias. Item response theory (IRT) parameters are mutually independent, and the parameters of an IRT model are said to be invariant to the targeted population. The underlying assumptions in IRT models include a confirmatory factor analysis model in which all items load on a single latent variable and the fact that each item’s uniqueness is uncorrelated (Luo et al., 2019). IRT provides information about person-by-item interactions, which are typically depicted by item characteristic curves (ICCs) (Embretson and Reise, 2004),namely, whether items measured some individuals more accurately than others. Measurement invariance can be examined through IRT in which latent variables are used to represent the construct to be measured by means of differential item functioning (DIF). DIF enables the variations of the item parameters across groups to be studied independently from additional parameters. The conformation of DIF exists based on the probability of providing a correct response between two groups that have similar abilities, indicating that the item measures an additional construct (Morán et al., 2018).

Although the advanced statistical methods (e.g., item response theory models) have been used numerous times for other scales (Chang et al., 2018), they have not been used to analyze the WBIS. Thus, the psychometric properties of the WBIS must be examined using advanced methods. Moreover, the *trans-*cultural utilization of foreign scales significantly differs from its utilization in the Chinese mainland on school-aged children and adolescents across weight categories. It is unwise to assess weight bias internalization if the scale is introduced merely based on previous studies without rewording sensitive items to mitigate its controversial aspects. Thus, this study aims to evaluate the reliability and validity of a Chinese version of the Weight Bias Internalization Scale (C-WBIS), and to select and validate C-WBIS items based on different psychometric analyses. According to Anna and Roberto’s findings (Roberto et al., 2012; Anna and Petra, 2018),the hypotheses of this study are as follows:

(1) The C-WBIS items would indicate an acceptable unidimensionality and yield reliable and valid scores for male and female children and in various age groups.
(2) The total score of the C-WBIS would be associated with higher levels of core self-evaluation and social anxiety.

## Materials and Methods

### Study Participants

The sample of this work comprised two subsamples and used the school class as a sampling unit. The first subsample comprised 200 female and 221 male primary and middle school students aged between 9 and 15 (*M* = 11.14 years; SD = 2.01 years) across weight categories. These participants were given a brief introduction and completed surveys under quiet conditions within the given 15-min period. There were 236 participants younger than 12 and 185 participants over 12. The second subsample included 43 students who were recruited for a student number to allow the matching of test–retest samples. Notably, the participation was voluntary. Consequently, the subjects’ decision to participate was made upon reading the informed consent document.

### Procedure

This study was approved by the Institutional Review Board of our university and provided all participants with a detailed overview before signing the written informed consent document. Parental informed consent and child assent were obtained from all subjects before data collection. Participation was voluntary and a notebook was provided as a reward for participation.

### Measures

Using the Weight Bias Internalization Scale (WBIS) for treatment-seeking obese adolescents (Roberto et al., 2012),the WBIS was translated from English into Chinese by three linguistic experts who highlighted uncertainties and challenging phrases through specific comments. The translated Chinese items were then examined by researchers. Then, a back translation was performed on items by another linguistic expert (who had no knowledge of the original English version of the WBIS) to identify discrepancies. This process ensured that the translation reflected the same item content as the original one. The C-WBIS was applied to a group of five students as a pilot application (these students would not participate in the final study) to collect information about any difficulties in completing the WBIS and to determine whether the purpose or meaning of each term could be accurately understood.

After minor revision, the C-WBIS was finalized. Specifically, we used the words “my weight” instead of the word “overweight” in evaluating the participants across different body weight statuses, and reworded controversial items that would cause additional distress to students. Thus, the item “My weight is a major way that I judge my value as a person.” was replaced with the item “My weight strongly influences what I think of myself confidence and worth as a person”(see Table 1). A total of 11 items were created and were rated on a seven-point Likert scale ranging from “Strongly Disagree” to “Strongly Agree,” to assess weight bias internalization, with higher values representing greater weight bias internalization.

**TABLE 1:** Original items of the WBIS and modified items of the C-WBIS.

**Original items (WBIS)**	**Modified items (C-WBIS)**
1. As an overweight person, I feel that I am just competent as anyone.	1. No matter how much I weigh, I can do just as much as everyone else
2. I am less attractive than most other people because of my weight.	2. I am less attractive than other people because of my weight
3. I feel anxious about being overweight because of what people might think of me	3. I feel anxious about my weight because of what people might think of me
4. I wish I could drastically change my weight	4. I wish I could change my weight a whole lot
5. Whenever I think a lot about being overweight, I feel depressed.	5. whenever I think a lot about my weight, I feel depressed
6. I hate myself for being overweight.	6. I hate myself because of my weight
7. My weight is a major way that I judge my value as a person.	7. My weight strongly influences what I think of myself confidence and worth as a person
8. I don’t feel that I deserve to have a really fulfilling social life, as long as I’m overweight.	8. Because of my weight, I don’t deserve having a lot of friends and fun
9. I am OK being the weight that I am.	9. I am satisfied with my weight
10. Because I’m overweight, I don’t feel like true self.	10. Because of my weight, I don’t feel like true self
11. Because of my weight, I don’t understand how anyone attractive would want to date me.	11. Because of my weight, I don’t understand why attractive peers would want to play with me.

Core self-evaluation was assessed using the Chinese version of the Core Self-Evaluation Scale (CSES) (Ren and Ye, 2009),which adopted a single dimension structure and comprised nine items (e.g., “I often feel depressed.”) that were rated on a five-point scale from one (Totally disagree) to five (Totally agree), with five items reverse scored. Higher scores indicated more frequent core self-evaluations. Cronbach’s alpha was satisfactory at 0.81.

Regarding the Social Anxiety Scale for Children (SAS) (La Greca et al., 1988), we employed the Chinese version of the Children’s Social Anxiety Scale to evaluate adolescents’ social anxiety (Li and Su, 2006). This scale consisted of ten items (e.g., “I worry about what other children say about me.”) rated on a three -point scale ranging from zero = never true to two = to always true, and was divided into two subscales: fear of negative evaluation (FNE) and social avoidance and distress (SAD). The higher the score, the greater the social anxiety of participants. In this study, Cronbach’s alpha coefficients were 0.71 for the FNE subscale and 0.68 for the SAD subscale.

Demographic information. Participants were asked about their gender and age using a demographic questionnaire.

### Item Selection and Validation

Based on the principles of item response theory (IRT) and classical test theory (CTT), the items of the C-WBIS were selected and validated.

### Classical Test Theory (CTT)

An item was considered for deletion if its standard deviation was ≤1, which evaluated the sensitivity of the items. Items were removed from the analysis if the corrected item-total correlation (CITC) was ≤0.40 (Ruzela et al., 2018). To estimate the internal consistency, the criterion for acceptable Cronbach’s alpha was a value of 0.70 or above when Cronbach’s alpha was computed. Test–retest assessment was completed (*n* = 43) using Pearson’s correlation coefficients. Test–retest intervals were set at 2 weeks.

To support the unidimensionality of the C-WBIS, we conducted a principal axis factor analysis using the first subsample (*n* = 421). If the ratio between the first and second eigenvalues exceeded three, a single latent construct was reflected (van der Linden and Hambleton, 1997). However, these methods may be inaccurate or subjective under a variety of conditions (Fabrigar et al., 1999). Parallel analysis is among the most accurate methods for identifying the correct number of factors by means of creating random datasets and extracting eigenvalues from the original dataset. The 95th percentile and mean of eigenvalues across the random datasets are computed. Then, the 95th percentile average eigenvalues are compared to the eigenvalues obtained from the original dataset via principal components analysis (Hayton et al., 2004). If factors with eigenvalues are greater than those of random datasets, they are retained (O’Connor, 2000).

The dimensionality and factor structure of the C-WBIS was assessed through a confirmatory factor analysis (CFA) to assess construct validity using AMOS 20.0. The goodness-of-fit criteria used in this study including the Comparative Fit Index(CFI) (good fit is ≥0.95), Tucker–Lewis Index(TLI) (good fit is ≥0.95), and root mean square error of approximation(RMSEA) (good fit is <0.06) were examined (Hu and Bentler, 1999). The criterion-related validity was confirmed through a correlation analysis between core self-evaluation and weight bias internalization and social phobia using Pearson’s correlation coefficients. We hypothesized that there was a correlation between the weight bias internalization, the core self-evaluation and social anxiety in moderate correlations (*r* = −0.30 −0.50) (Roberto et al., 2012; Hilbert et al., 2014b).

### Item Response Theory

Statistical analyses were performed using MULTILOG7.03 and WINSTEPS version3.72.3. The graded response model assessed item parameters using the marginal maximum likelihood estimation method (MML) to evaluate the following item parameters (van der Linden and Hambleton, 1997): discrimination (a), difficulty parameter (b). We then fitted the items with the graded response model (GRM),which provided estimates of marginal (i.e., aggregate) parameters that were most likely to have generated the observed sample data (Bock and Aitkin, 1982). Then, we assumed ordinal item responses and assessed item parameters using the MULTILOG7.03 software program (Stone, 1992), which detected a series of difficulty and discrimination parameters to select items. Items were selected when their discrimination estimate was <0.45 or the value of six degrees of difficulty (b1, b2, b3, b4, b5, and b6) that should be b1 < b2 < b3 < b4 < b5 < b6 was not in the range of -4 to 4 (Liao et al., 2012; Zhi et al., 2015). The graded-response model of IRT was suitable for polytomous items. Although, there was one item difficulty parameter in the dichotomous model, there were m-1 item difficulty parameters (Samejima, 1969),where m is the number of response categories (e.g., there are six b parameters for a seven-point scale). The probability of a participant responding to an item correctly was a function of two sets of parameters: person parameter and item parameters, which are typically depicted by item characteristic curves (ICCs) (Embretson and Reise, 2004). The *X*-axis indicates latent weight bias internalization in a standardized unit, and the *Y*-axis indicates the probability of choosing the number of response categories (Hulin et al., 1982).

Furthermore, Rasch analysis based on the Andrich rating scale model (joint maximum likelihood estimation) was used to detect differential item functioning (DIF), a method for studying measurement equivalence across groups which assesses the equivalence of both item discrimination and item difficulty parameters (Teresi and Fleishman, 2007). The significance of DIF might be identified from contrast that had been calculated using WINSTEPS software (version 3.72.3, Chicago, IL) (JM, 2005). This measure was used to assess DIF across different gender (female vs. male) and age (younger than 12 vs. over 12) groups that have different responses, despite having similar traits θ. A DIF value >0.5 logits is regarded as significant (Holland and Wainer, 1993; Morán et al., 2018). Thus the interpretation of the finding would be stratified by groups.

## Results

### Item Selection and Validation of the Scale

**FIGURE 1:**
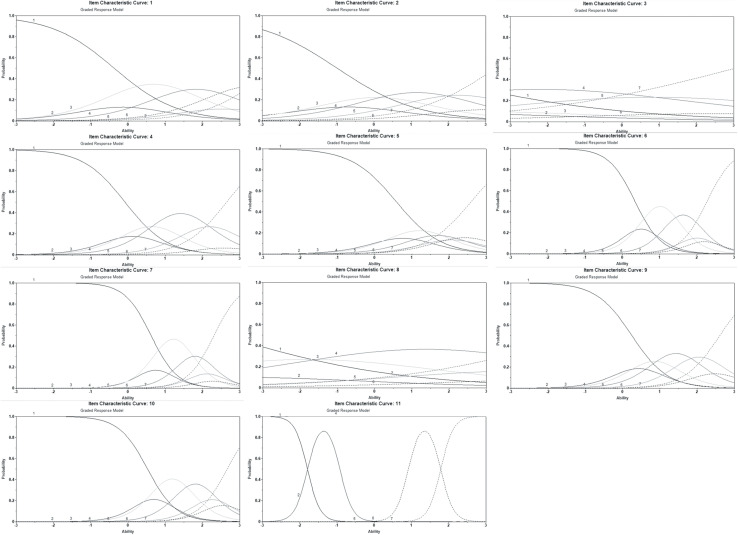
Matrix plot of item characteristic curves.

A combination of the results led to the removal of three items from the C-WBIS. In Table 2, Means, SDs, and item-total correlations are presented, for the 11 items that were included, and the values of item discrimination parameters (a) and the difficulty parameters (b) for each item were calculated for each item based on IRT. Items one and nine did not satisfy the condition of CITC ≤0.40, although their IRT results satisfied the condition of discrimination (a) and difficulty parameter (b). Ultimately, items one and nine were deleted. Figure 1 shows the matrix plot of item characteristic curves (ICCs) of IRT for the 11 items. The first panel presented the ICCs of items one to three for Mainland Chinese children and adolescents. The characteristic curve of three and eight should be deleted as these curves were not quite spread along the trait θ, and were “dense and clustered.” Item eight was retained because it was significantly close to the 0.45 threshold, while item three’s low discrimination led to its removal.

**TABLE 2:** Summary statistics of the C-WBIS.

**Item**	***M***	**SD**	**CITC**	**a**	**b1**	**b2**	**b3**	**b4**	**b5**	**b6**	**Selected items**
1. can do just as much^(r)^	3.13	1.68	**0.32**	1.18	−0.37	0.07	1.29	2.33	2.71	3.85	
2. Less attractive	2.44	1.54	0.53	0.94	−0.98	−0.42	0.55	1.72	2.77	3.23	√
3. Anxious about my weight	3.03	1.79	0.56	**0.37**	−5.91	−4.99	3.91	−0.47	2.10	2.93	
4. wish to change weight	4.67	1.87	0.41	1.77	−0.08	0.32	0.95	1.88	2.50	2.65	√
5. feel depressed	2.32	1.56	0.70	1.67	0.58	0.94	1.48	1.90	2.23	2.60	√
6. hate myself	1.98	1.65	0.53	2.84	0.34	0.68	1.36	1.90	2.11	2.28	√
7. Judgs elf worth as a person	1.86	1.36	0.66	2.82	0.62	0.87	1.58	2.02	2.22	2.31	√
8. Don’t deserve friends or fun	1.66	1.26	0.58	**0.43**	−4.11	−3.15	0.48	3.23	4.78	5.53	√
9. satisfied with my weight^(r)^	3.59	1.69	**0.38**	2.09	0.25	0.61	1.11	1.76	2.35	2.61	
10. Not feeling like true self	2.05	1.51	0.66	2.60	0.53	0.86	1.52	2.10	2.43	2.67	√
11. Not being dated	1.69	1.21	0.60	5.89	−1.79	−0.92	4.20	5.33	0.92	1.79	√

*The figures not meeting the statistical criteria are marked in bold, ^(*r)*^ reverse-scored; CITC = the corrected item-total correlation; a = Discrimination IRT parameter; b1, b2, b3, b4, b5, b6 = difficulty IRT parameters.*

**FIGURE 2:**
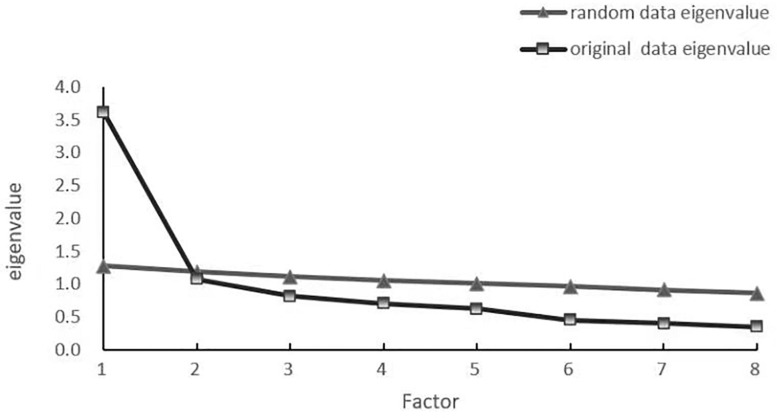
Plot of actual versus randomly generated eigenvalues.

Basic definitions and criteria were provided for each item parameter based on IRT and the principles of classical test theory (CTT), such as discrimination (a), difficulty parameters (b), and CITC. At this stage, it was decided to delete three items but to retain eight items. Thus, the analysis was run with the remaining eight items. As a prerequisite for conducting analysis based on IRT, a one-dimensional factor model needs to be confirmed. Principal components analysis (PCA) presented the C-WBIS as a single factor measure. The first eigenvalue (3.604) explained 45% of the variance, and the ratio of the first to the second eigenvalue was 3.6. A comparison of the original dataset eigenvalues and randomly generated eigenvalues suggested that the one-dimensional factor should be retained (see Table 3 and Figure 2 for eigenvalues). In the unidimensional model, the CFI, TLI, and RMSEA statistics were somewhat lower 0.954, 0.928, and 0.070 (90% CI = 0.049, 0.091).

**TABLE 3:** Actual and random eigenvalues from parallel analysis.

**Actual eigenvalue**	**Average eigenvalue**	**95th Percentile eigenvalue**
3.604	1.208	1.276
1.071	1.131	1.184
0.812	1.071	1.110
0.702	1.020	1.054
0.621	0.971	1.004
0.452	0.923	0.960
0.395	0.870	0.911
0.344	0.806	0.855

A requirement of the Rasch model is that the scale should establish measurement equivalence across different subgroups (e.g., age, gender), which could improve measurement precision. The table of DIF identification toward the overall items is displayed in the Table 4, indicating that no significant DIF was found across gender and age groups.

**TABLE 4:** Differential item functioning (DIF) for gender and age.

**DIF by gender**				**DIF by age**		
**Items**	**Man**	**Female**	**Contrast**	**<12**	**≥12**	**Contrast**
2	−0.19	−0.19	0.00	−0.19	−0.19	0.00
4	−1.56	−1.47	−0.09	−1.55	−1.47	−0.07
5	0.02	−0.24	0.26	−0.04	−0.18	0.14
6	0.34	0.21	0.14	0.28	0.28	0.00
7	0.47	0.53	−0.06	0.39	0.62	−0.23
8	0.39	0.54	−0.15	0.39	0.54	−0.14
10	0.08	0.13	−0.05	0.16	0.04	0.12
11	0.42	0.52	−0.10	0.54	0.40	0.14

### Correlations Among the Total C- WBIS, the CSES, and Two Subscales of the SAS

Associations were observed between weight bias internalization, core self-evaluation, and social anxiety. There were significant and moderate inverse correlations between core self-evaluation and weight bias internalization (*r* = −0.33, *p* < 0.01). Small but significant correlations were observed between C-WBIS and FNE (*r* = 0.22, *p* < 0.01) and SAD (*r* = 0.30, *p* < 0.01).

### Internal Consistency of the C- WBIS

In a nutshell, three items were removed based on the item response theory. The final eight-item C-WBIS showed acceptable internal consistency (Cronbach’s alpha = 0.79), and the test-retest reliability was satisfactory over 2 weeks (*r* = 0.81, *p* < 0.001).

## Discussion

This study evaluated the adaptation of the C-WBIS and found it to be a parsimonious and psychometrically valid and reliable scale among Mainland Chinese children and adolescent. Further, it is centered on the experience evaluation of weight bias and is relevant in a Chinese mainland context using Roberto’s WBIS (Roberto et al., 2012). Based on the principles of item response theory (IRT) and classical test theory (CTT), the items of the C-WBIS were selected and validated. CTT has its own strengths and weaknesses. It was easily understandable and provided school psychologists who might not be familiarity with IRT with psychometric information. However, the psychometric scale validation performance using CTT substantially depended on the examinee’s characteristics; that is, the person and item statistics were sample-dependent, which might engender different psychometric properties across different sample sizes (Abedalaziz et al., 2011). IRT was proposed on the basis of a single latent variable and also on the basis of the presence of local dependence (Luo et al., 2019). Thus, IRT and CTT can be combined to analyze the nature of the item characteristics (Abedalaziz et al., 2011). Meanwhile, modern psychometric techniques, represented by IRT, should be more widely used to have a greater degree of generalizability. The amount of the discrimination parameters in the C-WBIS ranged from 0.43 to 5.89 and provided an assessment of the sensitivity with larger discrimination values reflecting greater sensitivity.

Additional results supported the satisfactory reliability and validity of the unidimensional structure of the C-WBIS (eight-item scale with three items of the scale deleted) using IRT and CTT as a framework for investigating the item parameters. Additionally, the results were consistent with the aforementioned studies on adolescents and adults (Durso and Latner, 2008; Ciupitu-Plath et al., 2017). These findings were determined through parallel analysis, which suggested that one factor of the C-WBIS, based on factors with eigenvalues, was greater than those of random datasets. Further, CFA suggested that the C-WBIS was consistent with the data, which corroborates previous research indicating that the data-model fits the single-factor structure of the WBIS (Pakpour et al., 2019). Similar to Roberto et al. (2012), the reliability analysis demonstrated that the C-WBIS had an acceptable degree of internal consistency and test-retest reliability, which validated the stability of the WBIS-C’s scores among Mainland Chinese children and adolescents. Additionally, small positive correlations were found between C-WBIS and two subscales of the SAS, and an inverse relationship between CSES and C-WBIS was significant, which demonstrated an acceptable criterion validity. These findings were consistent with prior studies indicating an inverse relationship between CSES and C-WBIS (Hilbert et al., 2014b). Future research with an expanded sample size should explore CSES as a mediator of the relationship between weight bias internalization and social anxiety. It appears to involve a possible pathway toward understanding the development of social anxiety disorder.

Another aspect highlights measurement invariance as a key concept that had drawn wide attention. Our findings support the measurement invariance of the unidimensional factor model of gender groups. Thus, females and males participants in this study have equivalent meaning, and are consistent with a previous report examining German children, which contributes to the global use of this tool (Anna and Petra, 2018). Weight stigma leads to the weight bias internalization, which in turn causes higher levels of body shame (Tylka et al., 2014). In a Spanish study on 944 children aged between 9 and 12, Mendo-Lázaro et al. (2017) found that 57% (*n* = 538) of the participants wanted to become slimmer. Thus, we categorized the participants according to their age characteristics into two levels: younger than 12 and over 12. Our results showed that the measurement invariance of the single-factor structure was supported for C-WBIS across age (younger than 12 vs. over 12). These results expanded upon Roberto’s work and suggested that the C-WBIS would be culturally competent for children and adolescents in the Chinese mainland.

The findings of this study highlighted important issues related to the psychometric properties and acceptability of the C-WBIS. These findings suggest that analyses should be conducted to ensure that the environment, time, and process of the sample survey mitigated the disturbance to the survey as much as possible. Further, they indicate that the C-WBIS should be used regardless of whether adolescents’ weight status was categorized based on relative body mass index or self-reported, in that a high correlation pointed to a strong relationship between reported and measured Body Mass Index (BMI) (Haines et al., 2006). Lessons could perhaps be learned from the similarities between the reworded item that Anna and Petra (2018) highlighted regarding the acceptability of the measure. Similar to Hilbert et al. (2014b), we observed significant and moderate inverse correlations between core self-evaluation and weight bias internalization, which indicated that individuals with weight bias internalization were at risk of physical sub-health (Puhl and Latner, 2007). Contrary to our hypotheses, small but significant correlations between the C-WBIS score and two subscales of the SAS showed that increased weight bias internalization scores were correlated with a slight increase in social anxiety. This finding is at odds with research on individuals with comorbid social phobia, in which they were found to experience a higher rate of self-stigma (Vrbova et al., 2017).

Although the C-WBIS has shortcomings, every WBIS is technically deficient (Lee and Dedrick, 2016). Our results should, however, be interpreted with caution, although an advanced method was deliberately used to evaluate the psychometric properties of the C-WBIS. First, even though a sample size of 421 was sufficient to conduct IRT analyses, it was close to the minimum number required (Hulin et al., 1982). Specifically, the item parameters retrieved from a dataset were based on 421 Mainland Chinese children and adolescents across weight categories, and future studies seeking to collect information with a large sample size overcame the representativeness of the sample. Furthermore, the data were collected at a single point in time, and it is critical to carry out a longitudinal study to evaluate changes over time. Second, IRT analyses are affected by social desirability in that the desirability of the item might differ across cultures. Thus, future studies need to cautiously identify items indicating potential causes for improvement. The novelty of this research was that we created the C-WBIS, which can be utilized by male and female subjects of various age groups. We conducted a survey with homogeneous samples at a reasonable ratio for male and female participants, in each age span from 9 to 15 years.

The C-WBIS might be beneficial to interventions targeting weight stigma. With this tool, the comprehensive impact of treatment on weight stigma and its consequences (e.g., refusal to diet, binge eating) could be adapted to adolescents who might benefit from information on coping with weight stigma. School psychologists or social workers could help students adopt more reasonable coping strategies toward the consequences of weight stigma in their lives by advocating preventive efforts targeting children of different weights.

## Conclusion

In conjunction with existing research, this study finds that the C-WBIS, which has both psychometric and practical implications, has been adapted to assess weight bias internalization for Mainland Chinese children and adolescents across weight categories. This study supported a one-factor structure of the C-WBIS with eight items being defined more parsimoniously than in its original version. This study is novel in that it combines modern measurement theory, item response theory (IRT), with classical test theory (CTT) to assess the WBIS, which assesses weight bias internalization in the general population.

## Data Availability Statement

The raw data supporting the conclusions of this article will be made available by the authors, without undue reservation.

## Ethics Statement

The studies involving human participants were reviewed and approved by the Fujian Normal University Ethics Committee. Written informed consent to participate in this study was provided by the participants’ legal guardian/next of kin.

## Author Contributions

HC and Y-dY conceptualized the study. HC analyzed the data and wrote the manuscript. Y-dY supervised the whole study. Both authors contributed to the article and approved the submitted version.

## Conflict of Interest

The authors declare that the research was conducted in the absence of any commercial or financial relationships that could be construed as a potential conflict of interest.
